# Supplementary material for: Comparison of Fine-Needle Biopsy (FNB) versus Fine-Needle Aspiration (FNA) Combined with Flow Cytometry in the Diagnosis of Deep-Seated Lymphoma
Source: Diagnostics (Basel). 2023 Aug 28;13(17):2777. doi: 10.3390/diagnostics13172777 (PMC10487053; doi:10.3390/diagnostics13172777)
Supplement: Supplementary file 1 [file diagnostics-13-02777-s001.zip › Table S1.pdf]

Table S1. Stratification by lesion size – diagnostic rate

	FNB(n=23)	FNA(n=30)	OR (*95% CI*)	*P*-value
**< 20 mm，n**	**3**	**8**		
No. of cases consistent with final diagnosis by IHC, n (%)	3 (100.00%)	1 (12.50%)	8.000 (1.279 – 50.040)	0.024*
No. of cases consistent with final diagnosis by IHC+ FCM, n (%)	3 (100.00%)	7 (87.50%)	1.143 (0.880 – 1.485)	1.000
**≥ 20 mm，n**	**20**	**22**		
No. of cases consistent with final diagnosis by IHC, n (%)	18 (90.00%)	17 (77.30%)	2.647 (0.451 – 15.521)	0.414
No. of cases consistent with final diagnosis by IHC+ FCM, n (%)	20 (100.00%)	22 (100.00%)	-	-

* $P < 0.05$
