# Supplementary material for: Comparison of Fine-Needle Biopsy (FNB) versus Fine-Needle Aspiration (FNA) Combined with Flow Cytometry in the Diagnosis of Deep-Seated Lymphoma
Source: Diagnostics (Basel). 2023 Aug 28;13(17):2777. doi: 10.3390/diagnostics13172777 (PMC10487053; doi:10.3390/diagnostics13172777)
Supplement: Supplementary file 1 [file diagnostics-13-02777-s001.zip › Table S2.pdf]

Table S2. Diagnostic rate of Lymphoma Located in the Retroperitoneum

	FNB(n=20)	FNA(n=23)	OR (*95% CI*)	*P*-value
No. of cases consistent with final diagnosis by IHC, n (%)	19 (94.70%)	14(60.90%)	12.214 (1.383-107.865)	0.011*
No. of cases consistent with final diagnosis by IHC+ FCM, n (%)	20 (100.00%)	23(100.00%)	-	-

* $P < 0.05$.
