# Supplementary material for: Comparison of Fine-Needle Biopsy (FNB) versus Fine-Needle Aspiration (FNA) Combined with Flow Cytometry in the Diagnosis of Deep-Seated Lymphoma
Source: Diagnostics (Basel). 2023 Aug 28;13(17):2777. doi: 10.3390/diagnostics13172777 (PMC10487053; doi:10.3390/diagnostics13172777)
Supplement: Supplementary file 1 [file diagnostics-13-02777-s001.zip › Table S3.pdf]

Table S3. Univariate and multivariate logistic regression analysis –diagnostic rate combined with FCM

Variable	Univariate logistic regression			Multivariate logistic regression		
	Exp(b)	OR (95% CI)	*P*-value	Exp(b)	OR (95% CI)	*P*-value
Needle type (FNA vs FNB)	1.526	0.058-40.182	0.770	-	-	-
Needle size	1.460	0.280-7.622	0.605	-	-	-
Lesion site	4.626	0.918-26.493	0.051	1.091	1.008-1.180	0.038*
Lesion size	3.800	0.142-101.965	0.360	-	-	-
No. of lesions	-	-	-	0.920	0.826-1.024	0.122

* $P < 0.05$.
