# Supplementary figures and images for: Comparison of Fine-Needle Biopsy (FNB) versus Fine-Needle Aspiration (FNA) Combined with Flow Cytometry in the Diagnosis of Deep-Seated Lymphoma
Source: Diagnostics (Basel). 2023 Aug 28;13(17):2777. doi: 10.3390/diagnostics13172777 (PMC10487053; doi:10.3390/diagnostics13172777)

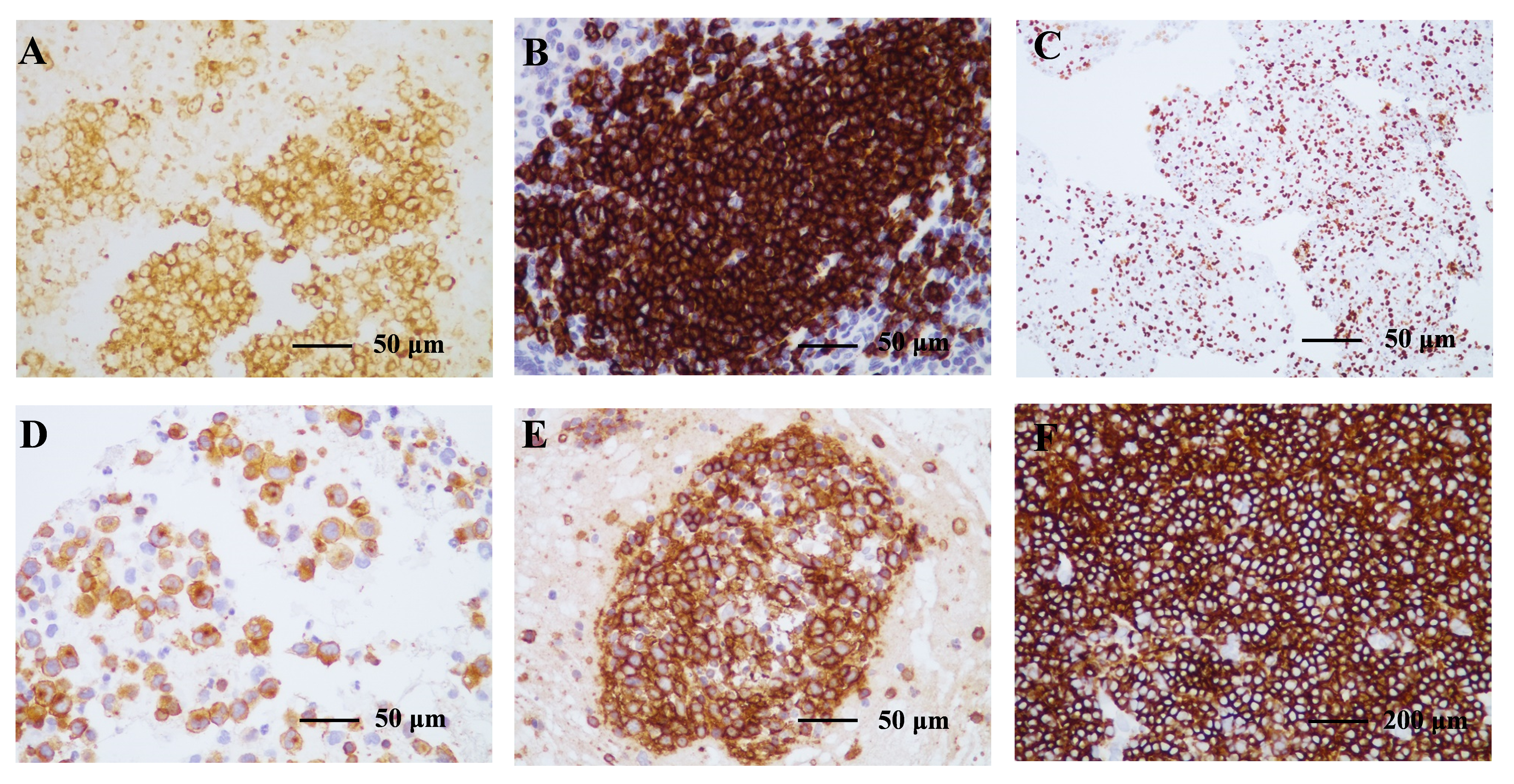

Supplement: Supplementary file 1 [file diagnostics-13-02777-s001.zip › Figure S1.tif]
